# Anemia and associated factors among 6 to 59 months age children attending health facilities in Kombolcha town, Northeast Ethiopia: a facility-based cross-sectional study

**DOI:** 10.1186/s12887-023-04031-z

**Published:** 2023-05-03

**Authors:** Wubshet Fentaw, Tefera Belachew, Assefa Andargie

**Affiliations:** 1 School of Public Health, College of Medicine and Health Sciences, Wollo University, Dessie, Ethiopia; 2 Department of Nutrition and Dietetics, Faculty of Public Health, Institute of Health, Jimma University, Jimma, Ethiopia; 3 Department of Public Health, College of Medicine and Health Science, Injibara University, Injibara, Ethiopia

**Keywords:** Anemia, Under-five children, Health facilities, Ethiopia

## Abstract

**Background:**

Childhood anemia is an important public health problem in Ethiopia. The northeast part of the country is among the areas affected by recurrent drought. Despite its significance, studies are scarce on childhood anemia particularly, in the study area. This study aimed to assess the proportion and factors associated with anemia among under-five children in Kombolcha town.

**Methods:**

A facility-based cross-sectional study was conducted among 409 systematically selected 6 to 59 months aged children visited health institutions in Kombolcha town. Data were collected using structured questionnaires from mothers/caretakers. The data entry and analysis were done using EpiData version 3.1 and SPSS version 26 respectively. Binary logistic regression was fitted to identify factors associated with anemia. Statistical significance was declared at p-value ≤ 0.05. The effect size was reported using the adjusted odds ratio with its 95% confidence interval.

**Results:**

Of the participants, 213(53.9%) were males with a mean age of 26 months (SD ± 15.2). The proportion of anemia was 52.2% (95% CI, 46.8-57%). Being in the age of 6–11 months (AOR = 6.23, 95% CI: 2.44, 15.95), 12–23 months (AOR = 3.74, 95%CI: 1.63, 8.60), having low dietary diversity score (AOR = 2.61, 95% CI: 1.55, 4.38), having a history of diarrhea (AOR = 1.87, 95% CI: 1.12, 3.12) and having the lowest family monthly income (AOR = 16.97, 95% CI: 4.95, 58.20) were positively associated with anemia. Whereas, maternal age ≥ 30 years (AOR = 0.37 (0.18, 0.77) and exclusive breastfeeding until six months (AOR = 0.27, 95% CI: 0.16, 0.45) were negatively associated with anemia.

**Conclusions:**

Childhood anemia was a public health problem in the study area. Child age, maternal age, exclusive breastfeeding, dietary diversity score, diarrhea, and family income were significantly associated with anemia.

**Supplementary Information:**

The online version contains supplementary material available at 10.1186/s12887-023-04031-z.

## Background

Anemia is a condition in which the hemoglobin concentration within the blood is lower than normal. The normal hemoglobin concentration varies by age, sex, elevation of residence, smoking habits and pregnancy status. Anemia among under-five children is defined as hemoglobin value less than 110 g/L [1]. The common causes of anemia in under-five children were found to be nutrient deficiencies, infectious diseases and genetic disorders [2, 3]. Iron deficiency is the most common cause of anemia and is estimated to contribute to approximately 42% of cases in children under 5 years of age worldwide [3].

Anemia is a major health problem throughout the world. The magnitude of the problem in developing countries is high since they are more exposed to various health and socioeconomic problems, which are directly or indirectly related to anemia. Though all age groups can develop anemia due to various factors, under-five children are among the most vulnerable age groups [4].

According to the 2019 estimation of the World Health Organization (WHO), 39.8% of under-five children in the world were anemic, of which 60.2% were in African region [5]. In East Africa, approximately 75% f under-five children have anemia with the prevalence ranging between 44% and 76% [6].As of the 2016 Ethiopian Demographic and Health Survey (EDHS), it was estimated that 57% of children under five years in Ethiopia had anemia with regional variation ranging from 42% in Amhara Region to 83% in Somali Region [7]. There are discrepancies in the prevalence of childhood anemia across studies conducted in different parts of the country. A study done in northeast Ethiopia (Guguftu) reported that 41.1% of under-five children were anemic [8]. Similarly, studies conducted in Gondar Town [9], North Shewa [10], Tigray [11], and Hawassa referral hospital [12] showed that the proportion of anemia among under-five children was 28.6%, 28.5%, 21.6% and 41.7% respectively.

The variation in the prevalence of anemia was attributed to multiplicity of factors. Childhood factors like age, residence, feeding practice, nutritional status, intestinal parasites, malaria, iron supplementation, vitamin A supplementation, deworming, eating raw foods, and dietary diversity were found to be associated with anemia. Moreover, familial and maternal factors including maternal age, educational status, marital status, family income, waste disposal practice, maternal anemia, household food insecurity, water source, family size, ante natal care, and maternal nutritional status were significantly associated with anemia [8–17].

Anemia among under-five children has serious consequences including growth retardation, poor immune system, and increased susceptibility to diseases and death, and has severe socio-economic consequences for families and communities [18–21]. It creates long-term effects among female children resulting in low-birth-weight babies and postpartum hemorrhage especially young children from low-income families have a higher risk for developing iron deficiency anemia [22]. Anemia is an indicator of both poor nutrition and poor health [5].

Several studies have been conducted across different parts of Ethiopia related to childhood anemia [8, 9, 14, 23–27]. Especially important is the study conducted in south Wollo Zone (Guguftu) which is located near to Kombolcha. The Guguftu study was conducted at a single public health center. In addition, the agro-ecological condition of Kombolcha is different from that of Guguftu. Kombolcha is a lowland area compared to Guguftu which is a highland ranging 3000–4000 m above sea level. This results in differences in agricultural products and general living condition of the society. Despite these and other studies, evidence is still scarce on childhood anemia in the Northeastern part of the country where drought and food insecurity are rampant in the area. Therefore, this study was conducted to determine the proportion and factors associated with anemia among under-five children visiting health facilities in Kombolcha town.

## Methods

### Study design and population

This study employed a facility-based cross-sectional design in Kombolcha town, Northeast Ethiopia. The study population was all children 6 to 59 months and visited selected public and private health facilities for any services in the town during the study period.

#### Study area and period

The study was conducted in Kombolcha town located in Amhara Regional State, South Wollo zone at a distance of 378 km from the capital Addis Ababa to the Northeast. The town is an important commercial and industrial center in northeast Ethiopia. The area is known for recurrent drought and least productivity. The study was conducted from February 15 to May 14, 2020.

### Eligibility criteria

All children aged 6 to 59 months who visited the selected health institutions were included in this study. Whereas, children with a known bleeding disorder, severe illness, mentally ill mothers or caretakers, and children who have taken blood and blood products (blood transfused) for the last 3 months were excluded from the study.

### Sample size and sampling procedure

The sample size was determined based on the single population assumption taking the prevalence of anemia to be 41.1% with a 5% margin of error and 95% confidence level [8]. By considering a 10% non-response rate the final sample size was estimated to be 409. The list of health institutions (4 Public health centers & 14 private medium clinics) was obtained from the town administration health office and was stratified into public and private. Then, half of the public and one-third of the private institutions were selected using a simple random sampling technique (lottery method). The study subjects were selected using a systematic sampling method. The first child was selected by simple random sampling (lottery method) and then every 2 children was selected based on the order of their visit. Systematic random sampling can be applied in the absence of a frame for the population [28].

### Operational definitions

#### Anemia

In children under five years, anemia was defined as a hemoglobin level below 11 g/dl. In this study, the Hgb level was adjusted to the altitude. Since the study area is found 1842 m above sea level, 0.8 g/dl was added to the standard Hgb cut-off value. Therefore, children with an Hgb of 11.8 g/dl or more were considered normal while children who had an Hgb value *<* 11.8 g/dl were considered anemic [7]. This cut-off point works for both male and female children since there is no gender variation until 12 years of age [2, 29].

#### Dietary diversity score

this is determined by counting the number of food groups consumed in the last 24-hours. Consumption of 5 to 8 and 4 to 7 food groups were considered adequate dietary diversity for children 6 to 23 and 24 to 59 months respectively [30].

#### Caretaker

the person who is responsible for parenting the child. This person can be the biological parents (mother or father), any member of the family or anyone not a member of the family responsible for provision of care and support for the child.

### Data collection tools and procedures

Pretested and structured questionnaires adapted from previous similar pieces of literature [8, 14, 26, 31–36] were used to collect relevant data for this study. The first part of the questionnaire included the socioeconomic and demographic characteristics of the child and the mother/caretaker. The average monthly family income was determined by asking the caretakers to recall the estimated monthly income in Ethiopian currency. The second part included common medical comorbidities pertaining to the child intended to recall the presence of history of malaria, TB, HIV, diarrhea and vitamin A supplementation in the last six months. The third part was a questionnaire on the individual dietary diversity score. The questionnaire was adopted from the FAO/FANTA guidelines for measuring household and individual dietary diversity [37] and WHO IYCF guideline [30] which are based on the 24-hour free recall approach. In addition, respondents were asked to indicate whether or not their child consumed any food over the previous 24 h from each of eight food groups for children 6 to 23 months and seven food groups for children 24 to 59 months. The questionnaire was prepared in English, translated into the local language, and then back to English by English language experts to ensure its consistency. Six trained nurses and six laboratory technicians conducted the data collection.

#### Blood specimen collection and examination

After asking about the willingness of parents or caregivers, capillary blood samples were collected from children under strict aseptic precautions by trained laboratory technicians. The hemoglobin concentration was measured using HemoCue Hb 201 analyzer.

#### Stool specimen collection and examination

A clean plastic container marked with an identification number was used to collect about 2 mg of stool sample from each child. Then, a stool wet mount smear was prepared using saline and/or iodine solution for direct microscopic identification of intestinal parasites within 30 min of sample collection. The direct smear was examined first by 10x and then 40x objective for detection of helminth eggs, larvae, and protozoan parasites by experienced medical laboratory technicians.

### Data quality control

The quality of data was assured through careful designing, translation, pre-testing of the questionnaire, and training of data collectors. Every day after data collection, the filled questionnaire was checked for completeness by the principal investigator, supervisors, and corrections were made accordingly for the next day. Standard operating procedures and manufacturers’ instruction was strictly followed for all laboratory activities. All laboratory reagents were checked for their expiry date. Samples were checked whether they are in the acceptable criteria like volume, consistency, and collection time. Microscopic slides and cover glasses were checked for cleanliness.

### Data processing and analysis

The collected data were entered into a controlled data entry template using Epidata version 3.1 and exported to SPSS version 26.0 for analysis. Exploratory analysis was performed to check missing values, outliers and assumptions before the main analysis was carried out. Descriptive statistics like frequencies, proportions, and summary measures were computed. Binary logistic regression was employed to identify factors associated with anemia. First bivariate binary logistic regression was performed and variables found to be significant at P-value < 0.25 were imputed into multiple logistic regression. A p-value *<* 0.05 was used to indicate statistical significance in the multiple logistic regression model. Both the crude and adjusted Odds Ratios were reported as effect measures. The results were presented using tables, figures, and text narratives.

## Results

### Characteristics of study participants

Out of 409 participants, 395 participants responded correctly making the response rate 96.6%. About 213(53.9%) children were males with a mean age of 26 months (SD ± 15.2). Of the total mothers interviewed, 211 (53.4%) attended secondary education and above; about 330 (83.5%) were from urban residences. Regarding the marital status of mothers, 357 (90.3%) were married (Table 1).

**Table 1:** Socio-demographic characteristics of children and mothers/caretakers attending health institutions of Kombolcha town, Northeast Ethiopia (n = 395)

Variables		Frequency	Percent
Child Sex	Male	213	53.9
	Female	182	46.1
Child age	6–11	72	18.2
	12–23	128	32.4
	24–35	80	20.3
	36–47	57	14.4
	48–59	58	14.7
Residence	Urban	330	83.5
	Rural	65	16.5
Religion	Orthodox	124	31.4
	Muslim	265	67.1
	Protestant	6	1.5
Maternal age in years	18–27	219	55.4
	28–38	168	42.5
	>=39	8	2.1
Marital status of the mother	Single	29	7.3
	Married	357	90.4
	Divorced	9	2.3
Ethnicity	Amhara	374	94.7
	Tigre	17	4.3
	Other	4	1.0
Educational status of mothers	No formal education	37	9.4
	Primary education	147	37.2
	Secondary education and above	211	53.4
Mothers’ occupation	Housewife	304	77.0
	Employed	81	20.5
	Student	10	2.5
Monthly family income (ETB)	< 1500	62	15.7
	1501–3000	67	17.0
	3001–4500	201	50.9
	> 4500	65	16.5
Family size	< 4	158	40.0
	4 and above	237	60.0

**Abbreviations:** ETB, Ethiopian Birr

### Nutrition and disease morbidities

Out of 395 study subjects, 171 (43.3%) had diarrhea of which 102 (49.8%) were anemic. Most of the 256 (64.2%) children got adequate dietary diversity scores (four and more food groups) per 24 h out of them 104 (40.6) were anemic (Table 2).

**Table 2:** Characteristics related to nutrition and morbidity among children under-five years attending health facilities in Kombolcha town, Northeast Ethiopia (n = 395)

Variables	Category	Frequencies	Percent
History of malaria in children	Yes	14	3.5
	No	381	96.5
History of TB	Yes	2	0.5
	No	393	99.5
Vitamin A supplements and deworming	Yes	146	37
	No	249	63
Diarrhea in children	Yes	171	43.3
	No	224	56.7
Exclusive breastfeeding	No	222	56.2
	Yes	173	43.8
Dietary diversity score	Low	193	48.9
	Adequate	202	51.1

**Abbreviation:** TB, Tuberculosis

### Anemia among children

**Fig. 1:**
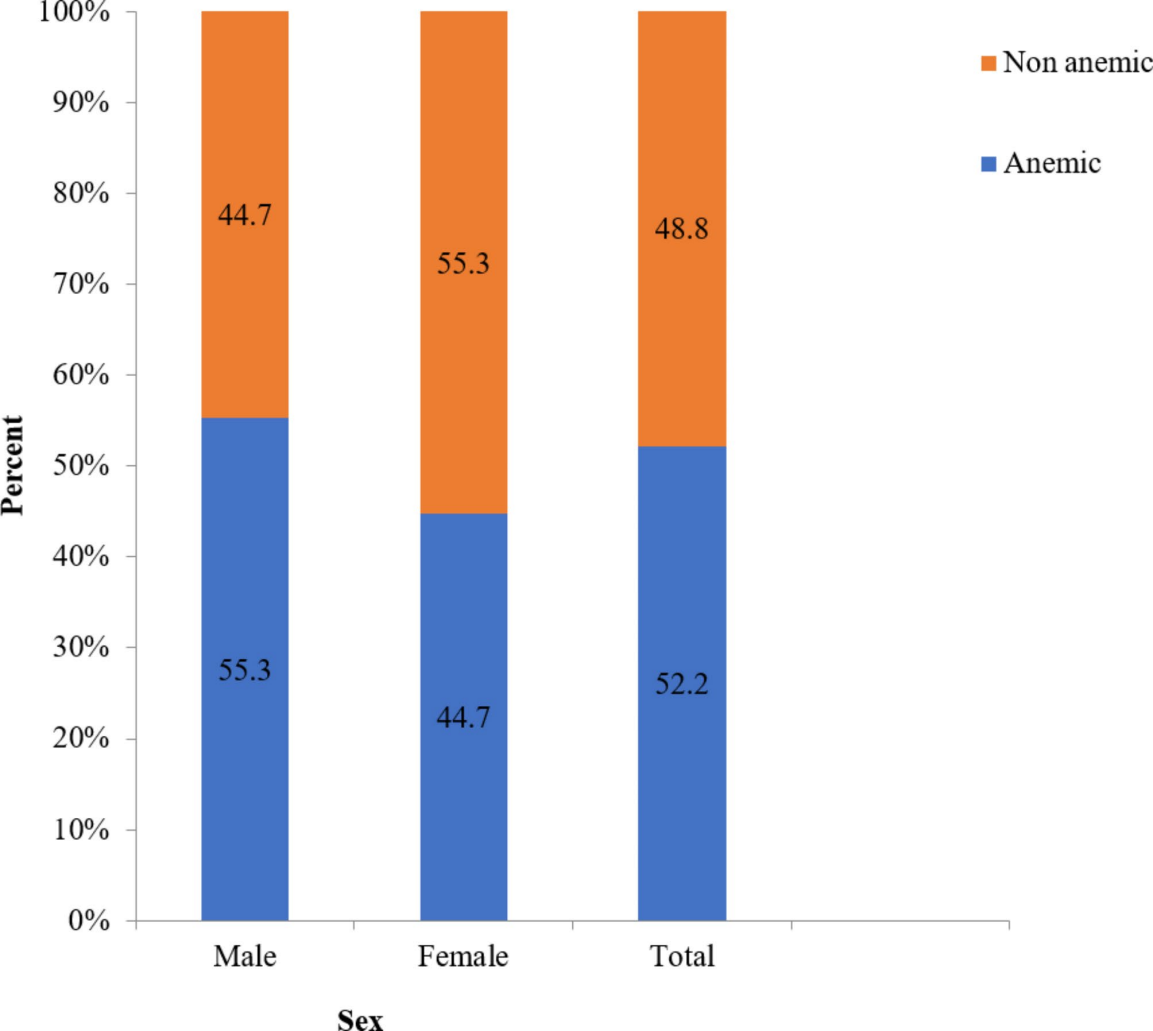
Proportion of anemia aggregated by child sex

The mean hemoglobin value of children was 11.4 g/dl (SD ± 18.3). Of all children, 206 (52.2%; 95% CI 46.8–57.0) were anemic, of which 114 (55.3%) were males (Fig. 1).

### Factors associated with anemia

Children in the age group of 6–11 months were six times more likely to have anemia as compared to 48–59 months (AOR: 6.23, 95% CI: 2.44, 15.95). Similarly, children 12–23 months of age were 3.74 times as likely to be anemic than 48–59 months children (AOR: 3.74, 95% CI: 1.63, 8.60). Children from mothers of at least 30 years old were 63% less likely to be anemic as compared to children from younger mothers (AOR: 0.37, 95% CI: 0.18, 0.77). Children who exclusively breastfed up to six months were 73% less likely to be anemic compared to children who were not exclusively breastfed (AOR: 0.27, 95% CI: 0.16, 0.45). Children having low dietary diversity scores were 2.6 times more likely to have anemia than those having adequate dietary diversity scores (AOR: 2.61, 95% CI: 1.55, 4.38). Children who have a history of diarrhea in the past 2 months before the data collection were nearly 2 times more likely to be anemic than children who do not have a history of diarrhea (AOR: 1.87, 95% CI: 1.12, 3.12). Children from households with a family monthly income of < 1,500 ETB were 17 times more likely to have anemia as compared to those who had a family income of > 4,500 ETB (AOR: 16.97, 95%CI: 4.95, 58.20) (Table 3).

**Table 3:** Factors associated with anemia among children 6–59 months in Kombolcha town, Northeast Ethiopia (n = 395)

Factors		Anemic (Count (%))	Not anemic (count (%))	COR (95% CI)	AOR (95% CI)	P-value
Age of Children (in months)	6–11	51(70.8)	21(29.2)	6.38 (2.9, 13.7)	6.23 (2.44, 15.95)	**< 0.001**
	12–23	76(59.4)	52(40.6)	3.84 (1.9, 7.5)	3.74 (1.63, 8.60)	**0.002**
	24–35	36(45.0)	44(55.0)	2.14 (1.04, 4.4)	2.31 (0.94, 5.68)	0.067
	36–47	27(47.4)	30(52.6)	2.36(1.08, 5.1)	2.44 (0.93, 6.44)	0.071
	48–59	16(27.6)	42(72.4)	1.00	1.00	
Residence	Urban	159(48.2)	171(51.8)	1.00	1.00	
	Rural	47(72.3)	18(27.7)	2.81 (1.57, 5.04)	1.89 (0.85, 4.20)	0.119
Maternal age (in years)	< 30	182(55.5)	146(44.5)	1.00	1.00	
	>=30	24(35.8)	43(64.2)	0.45 (0.26, 0.77)	0.37 (0.18, 0.77)	**0.008**
Exclusive Breast Feeding (EBF)	No	147(66.2)	75(33.8)	1.00	1.00	
	Yes	59(34.1)	114(65.9)	0.26 (0.17, 0.40)	0.27 (0.16, 0.45)	< **0.001**
Dietary Diversity Score	Low	134(69.4)	59(30.6)	4.10 (2.69, 6.24)	2.61 (1.55, 4.38)	< **0.001**
	Adequate	72(35.6)	130(64.4)	1.00	1.00	
Diarrhea in the last 2 months	No	104(46.4)	120(53.6)	1.00	1.00	
	Yes	102(59.6)	69(40.4)	1.71 (1.14, 2.55)	1.87 (1.12, 3.12)	**0.017**
Monthly income (ETB)	< 1,500	58(93.5)	4(6.5)	18 (5.8, 55.4)	16.97 (4.95, 58.20)	< **0.001**
	1,501-3,000	43(64.2)	24(35.8)	2.2 (1.1, 4.4)	1.61 (0.67, 3.87)	0.283
	3,001–4,500	76(37.8)	125(62.2)	0.75 (0.4, 1.3)	0.64 (0.33, 1.25)	0.190
	> 4,500	29(44.6)	36(55.4)	1.00	1.00	

**Abbreviations:** AOR, Adjusted Odds Ratio; CI, Confidence Interval; COR, Crude Odds Ratio; DDS, Dietary Diversity Score; EBF, Exclusive Breast Feeding; ETB, Ethiopian Birr

## Discussion

This study was conducted to determine the prevalence of anemia and associated factors among children 6 to 59 months in Kombolcha town, northeast Ethiopia. The proportion of children with anemia was found to be 52.2% (95%CI = 47.2%, 57.1%), which is considered a severe public health problem based on the WHO severity classification of anemia (> 40%) [38]. This finding is comparable with findings in Southern Ethiopia [39] and Brazil [40]. However, it is higher than findings from Guguftu (Northeast Ethiopia) [8], Gondar (Northwest Ethiopia) [9], and Hawassa (Southern Ethiopia) [27]. This discrepancy may be justified by the fact that the area, where this study was conducted, is frequently affected by drought and conflict incidents so there might be food insecurity [41]. According to the 2019 and 2020 analysis of the Integrated Food Security Phase Classification (IPC), the north eastern part of Ethiopia was classified in Phase 2 (stressed) and above food insecurity level [42, 43]. The seasonal variation of the studies may also explain the difference. However, this finding is lower than findings from Bedele Hospital (Southwest Ethiopia) [44], and Tanzania [45], The Bedele study was conducted among children admitted to the hospital. It seemed those children may have severe/complicated condition that made them to be admitted in the hospital. Even some of them may be admitted due to anemia and related conditions. These reasons may precipitate anemia and increase its proportion. The Tanzanian study was conducted in the community among rural children. Rural residence is one factor that increases the prevalence of anemia as evidenced by literatures [46]. In addition, the current prevalence of anemia is lower than the national anemia prevalence in children under five years of age. As Kombolcha is found in the Amhara region, the region had the lowest prevalence of anemia in the country [7].

Younger children were at a higher risk of anemia than older children. This finding is supported by studies in Guguftu (Northeast Ethiopia) [8], Gondar town (Northwest Ethiopia) [9], Adami Tullu (South Central Ethiopia) [47], Hawassa (Southern Ethiopia) [27] and Tanzania [45]. Infants began to eat complementary foods after six months old. From this time onwards up to 2 years there might be difficulty in ingesting such complementary feed and may result in a deficiency of nutrients leading to a higher probability of anemia. On contrary, children would have the ability to eat a variety of foods when they become older.

The age of the mother was significantly associated with anemia. Children born from older mothers were less likely to be anemic compared to children from younger mothers. This is in line with a study in Ghana [48]. As the maternal age increases, the mother probably will have more children so a better experience in child care and feeding. The better the care and feeding practice a child gets, the lesser risk of malnutrition.

Whether a child was exclusively breastfed or not also matters the occurrence of childhood anemia. Children who were exclusively breastfed until six months are less likely to be anemic than their counterparts. This finding contradicted a study in China [33]. This discrepancy may be explained by the socio-economic differences in the study participants and study design. Exclusive breastfeeding for the first six months is a highly recommended feeding practice by the WHO. It provides the optimal feeding option by preventing diarrheal diseases, respiratory tract infections, and chronic diseases [49]. This may help exclusively breastfed children to be at a lower risk of anemia.

The dietary diversity score of a child was associated with anemia. Children who had low dietary diversity scores were more likely to be anemic than children who had adequate dietary diversity scores. This finding is similar to a study done in Hohoe Municipality, Ghana [50]. However, there was no significant association between DDS and anemia among studies conducted in Menz Gera (Central Ethiopia) [10], and Kilte Awulaelo (Northern Ethiopia) [26]. Eating a variety of foods may provide all the necessary nutrients, prevent disease, and boost the immunity of the child. Hence, a child with an adequate dietary diversity score may have a low risk of anemia.

The occurrence of anemia was not the same among children who had diarrhea and no diarrhea. The odds of anemia among children who had diarrhea for the last two months before the date of the interview were almost two times higher than their counterparts. This finding contradicted a study in the University of Gondar specialized hospital (Northwest Ethiopia) [14]. This contradiction can be explained by the difference in the study participants. The current study was conducted among ambulatory patients while the study in the University of Gondar specialized hospital was conducted among admitted pediatric patients. On the other hand, the University of Gondar hospital study considered acute diarrhea so that there might be a decrease in body fluid and mask the low level of hemoglobin value. Chronic diarrhea results the depletion of important nutrients including iron due to its poor absorption in the gastrointestinal tract. But diarrhea had no association with anemia among studies conducted in Gondar (Northwest Ethiopia) [9] and Tigray (Northern Ethiopia) [11].

As family monthly income increased, the prevalence of anemia decreased markedly similar to the findings in the Tigray region (Northern Ethiopia) [51], Bedele Hospital (Southwest Ethiopia) [44], Guguftu Health Center (Northeast Ethiopia) [8] and Sudan [36]. The relationship between socioeconomic status and malnutrition is well established. Low socioeconomic status may result in food scarcity, poor hygiene, and poor child care in general. This in turn may precipitate the occurrence of malnutrition including iron deficiency anemia.

The result of this study indicated that anemia is a major public health problem among children under-five years attending health institutions in Northeast Ethiopia. According to the WHO classification for persistent anemia in a population (40%) and above, this finding confirmed that anemia among under-five children was a severe public health problem. Such a high magnitude of anemia among under-five children corroborates the finding of EDHS affirming the fact that despite the implementation of micronutrient deficiency prevention and control guidelines for several years. Endorsement and implementation of the national nutrition strategy and the greater emphasis given to anemia in the newly endorsed food and nutrition policy, there remains much to be done to cure the problem of anemia. The findings call for a galvanizing community-level intervention that addresses key behaviors such as breastfeeding and dietary behaviors.

### Limitations of the study

Anemia was measured based on only hemoglobin concentration parameters, the inclusion of other measures such as serum ferritin could lead to a better differentiation of cases. Being an institution-based study, the result cannot be extrapolated to the larger community. The presence of comorbidities like malaria, TB, HIV and diarrhea were not confirmed by objective methods. We only asked the caretakers to respond whether their child was encountered with these conditions in the last six months. The certainty of this response would be affected by the recall and literacy of respondents. Moreover, the study was done only in one season and may not represent the magnitude year-round.

## Conclusions

The burden of anemia among children aged 6–59 months in the study site was high and it has severe public health problems according to the WHO cut-off points. The age of the child, the age of the mother, family monthly income, exclusive breastfeeding status, and having a low dietary diversity score are significantly associated with anemia. The healthcare workers are advised to educate the pregnant and lactating mothers, and the whole community about good dietary practices, the benefit of exclusive breastfeeding, and income-generating activities by collaborating with the finance and economy development sectors. Researchers are recommended to use additional biomarkers to differentiate the cause of anemia and its seasonality using longitudinal designs. Moreover, studies aimed at measuring the effect of the covid-19 and the conflict on childhood anemia are recommended so as to produce evidences to achieve resilient health system.

## Electronic supplementary material

Below is the link to the electronic supplementary material.

Supplementary Material 1

## Data Availability

All data generated or analyzed during this study are included in this published article and its supplementary information files.

## List of Abbreviations

ANC: Ante Natal Care
AOR: Adjusted Odds Ratio
CI: Confidence Interval
COR: Crude Odds Ratio
DDS: Dietary Diversity Scores
EBF: Exclusive Breastfeeding
EDHS: Ethiopian Demographic and Health Survey
ETB: Ethiopian Birr
G/dl: Gram per deciliter
HC: Health center
HCT: Hematocrit
Hgb: Hemoglobin
HIV: Human Immunodeficiency Virus
mg: Milligram
OR: Odds Ratio
SD: Standard Deviation
SPSS: Statistical Product and Service Solutions
TB: Tuberculosis
UN: United Nation
WHO: World Health Organization
